# Clinical and metabolic links between hypothyroidism and metabolic syndrome: a review

**DOI:** 10.3389/fendo.2026.1890732

**Published:** 2026-09-18

**Authors:** Mubai Li, Xiaozhu Guo, Na Ren, Ting Wang, Huihui Zhu, Haixin Zhang, Chang Liu

**Affiliations:** 1 Heilongjiang University of Chinese Medicine, Harbin, China; 2 Hangzhou Fang Rejuvenation Hall Xiasha Traditional Chinese Medicine Outpatient Department Co., Ltd, Hangzhou, China

**Keywords:** dyslipidemia, hypothyroidism, insulin resistance, metabolic syndrome, obesity

## Abstract

Hypothyroidism and metabolic syndrome frequently coexist and share abnormalities in body weight, lipid metabolism, glucose regulation, and inflammatory signaling. Thyroid hormone deficiency can contribute to dyslipidemia, reduced energy expenditure, and impaired insulin action. By contrast, evidence that obesity, dyslipidemia, or insulin resistance directly causes thyroid dysfunction remains less established and is derived mainly from observational and experimental studies. This critical narrative review integrates current mechanistic and clinical evidence across obesity, dyslipidemia, and insulin resistance. It also examines implications for diagnosis, thyroid hormone replacement, metabolic treatment, monitoring, and future research. Clinical management should restore euthyroidism when thyroid treatment is indicated and address persistent obesity, dyslipidemia, and abnormal glucose metabolism according to their respective clinical indications. Longitudinal and interventional studies are needed to clarify causal direction and identify patient subgroups that may benefit from more individualized management.

## 1 Introduction

Hypothyroidism and metabolic syndrome are common endocrine and metabolic disorders that frequently coexist and are both associated with increased cardiovascular risk (1–3). Their clinical overlap is important because thyroid hormone deficiency can affect energy expenditure, lipid metabolism, and insulin sensitivity. At the same time, metabolic abnormalities may influence thyroid homeostasis through neuroendocrine, inflammatory, and peripheral metabolic pathways (4, 5). Epidemiological studies have reported a higher prevalence of metabolic syndrome among patients with hypothyroidism and an increased occurrence of subclinical hypothyroidism in individuals with metabolic syndrome (6–9).

Despite this clinical overlap, several important uncertainties remain. Existing studies have often examined individual pathways or isolated metabolic components, while the strength and direction of reverse metabolic effects on thyroid function remain less clearly established. The clinical significance of these associations is also uncertain, particularly when mechanistic findings are translated into decisions on screening, treatment intensity, and long-term monitoring. These limitations create a gap between the growing mechanistic literature and its application to the management of patients with both conditions.

This critical narrative review therefore integrates current mechanistic and clinical evidence across obesity, dyslipidemia, and insulin resistance. It examines how hypothyroidism is associated with these metabolic abnormalities and evaluates evidence that metabolic disturbances may in turn influence thyroid function. The review also considers the implications of these relationships for clinical assessment and treatment. Particular attention is given to thyroid status and the degree of thyroid-stimulating hormone (TSH) elevation when metabolic risk is interpreted (10, 11). Areas of inconsistent evidence and priorities for future research are then discussed. By distinguishing established associations from less certain causal interpretations, this review aims to provide a clearer framework for clinical management and further investigation.

## 2 Review approach

This article was developed as a critical narrative review. Relevant literature was identified through targeted searches of PubMed and Web of Science from database inception through August 2026. The main search concepts included hypothyroidism, subclinical hypothyroidism, metabolic syndrome, obesity, dyslipidemia, insulin resistance, thyroid-stimulating hormone, thyroid hormone, and levothyroxine. Recent clinical guidelines, systematic reviews, meta-analyses, and human clinical studies were prioritized when epidemiological associations and clinical management were evaluated. Earlier landmark studies were retained when they provided foundational evidence for established physiological or molecular mechanisms. Animal and cellular studies were used primarily to interpret mechanistic pathways when corresponding human evidence was limited.

Literature identified through the targeted searches was considered for inclusion when it directly informed one or more of the principal clinical or mechanistic domains addressed in this review. Evidence was integrated according to its relevance to the principal metabolic domains, with particular attention to inconsistent findings and unresolved clinical questions. This approach was intended to provide a clinically oriented narrative synthesis and was not designed as a systematic review or meta-analysis. Representative clinical studies were selected purposively from the human studies included in the narrative synthesis to illustrate the principal clinical questions addressed in the review. Selection emphasized studies that provided direct and clinically interpretable evidence on major clinical associations, including cardiovascular risk (11). Studies addressing physiological mechanisms and metabolic outcomes were also considered when they provided clinically relevant evidence (12). Interventional evidence was included when it helped define the effects and limitations of treatment in clinically relevant populations (13). The selection also sought to represent different study designs and areas in which the available evidence remained uncertain or heterogeneous. The studies summarized in **Tables 1**, **2** are therefore intended as a focused illustration of the clinical evidence rather than a comprehensive list of all studies identified in the targeted searches.

**Table 1:** Representative clinical studies illustrating the major themes of the narrative synthesis.

Study	Design and population	Main finding	Relevance to the review
Rodondi et al. (11)	Individual-participant analysis of 11 prospective cohorts including 55,287 adults	CHD event risk increased across higher TSH categories, with the strongest association at TSH 10-19.9 mIU/L	Supports graded cardiovascular risk interpretation in subclinical hypothyroidism
Karmisholt et al. (14)	One-year controlled follow-up of 12 newly diagnosed hypothyroid patients and 10 euthyroid controls	LT4-associated weight loss occurred without a corresponding reduction in fat mass	Distinguishes loss of excess body water from adipose-tissue loss after treatment
Maratou et al. (12)	Clinical physiological study including 12 patients with overt hypothyroidism, 13 with subclinical hypothyroidism, and 21 euthyroid controls	Hypothyroid groups showed higher HOMA-IR and reduced insulin-stimulated GLUT4 translocation and glucose transport	Supports an association between hypothyroidism and impaired peripheral insulin action
Severo et al. (15)	Randomized double-blind placebo-controlled trial of 48 adults with subclinical hypothyroidism	Metformin did not demonstrate a consistent reduction in TSH compared with placebo	Does not support metformin as a thyroid-directed treatment
Zijlstra et al. (13)	Pooled individual data from two randomized placebo-controlled trials including 842 older adults with subclinical hypothyroidism	LT4 did not significantly reduce cardiovascular events, atrial fibrillation, heart failure, or all-cause mortality	Demonstrates uncertainty regarding cardiovascular benefit of LT4 in older adults with subclinical hypothyroidism
Yang et al. (16)	Cross-sectional study including 622 adults with newly diagnosed type 2 diabetes and 621 normoglycemic adults	Subclinical hypothyroidism was associated with greater insulin resistance in normoglycemic participants but not in those with type 2 diabetes	Indicates that the thyroid-insulin resistance association varies according to metabolic context
Ostadrahimi et al. (17)	Before-and-after study of 105 untreated patients with primary hypothyroidism receiving LT4 for three months	Lipid parameters and fasting insulin improved, while HOMA-IR and QUICKI did not change significantly	Shows that metabolic responses to restoration of euthyroidism are not uniform
Zhao et al. (18)	Population-based case-control study involving 24,100 participants	Hypertriglyceridemia was associated with higher odds of subclinical hypothyroidism	Supports an observational lipid-to-thyroid association without establishing causal direction

CHD, coronary heart disease; GLUT4, glucose transporter type 4; HOMA-IR, homeostatic model assessment of insulin resistance; LT4, levothyroxine; QUICKI, quantitative insulin sensitivity check index; TSH, thyroid-stimulating hormone.

**Table 2:** Qualitative evidence appraisal of the representative clinical studies summarized in **Table 1**.

Study	Design strength	Main limitation	Interpretation in this review
Rodondi et al. (11)	Large individual-participant dataset with prospective cardiovascular follow-up	Observational design remains susceptible to residual confounding and between-cohort heterogeneity	Supports risk association and TSH-gradient interpretation but not treatment causality
Karmisholt et al. (14)	Longitudinal assessment with direct body-composition measurements	Small sample and nonrandomized treatment exposure	Supports the contribution of fluid loss to treatment-related weight change but not population-level estimates
Maratou et al. (12)	Combined *in vivo* and cellular assessment of insulin action	Small groups and cross-sectional comparisons	Provides physiological support for impaired insulin action but cannot establish long-term clinical causality
Severo et al. (15)	Randomized double-blind placebo-controlled design	Small sample and short follow-up with TSH as the principal thyroid outcome	Provides direct interventional evidence but limited precision for longer-term thyroid outcomes
Zijlstra et al. (13)	Randomized placebo-controlled data with clinically relevant cardiovascular outcomes	Restricted to older adults with subclinical hypothyroidism and relatively few cardiovascular events	Stronger evidence for older adults but limited generalizability to younger patients or higher TSH ranges
Yang et al. (16)	Large clinically characterized population with separate glucose-status groups	Cross-sectional design limits temporal and causal interpretation	Supports metabolic-context heterogeneity rather than a fixed causal relationship
Ostadrahimi et al. (17)	Prospective measurement before and after restoration of thyroid function	No untreated comparison group and relatively short follow-up	Supports improvement in selected metabolic markers but not consistent reversal of insulin resistance
Zhao et al. (18)	Large population-based sample with matching and adjustment for potential confounders	Case-control design cannot exclude residual confounding or reverse causation	Supports an epidemiological association while the lipid-to-thyroid pathway remains hypothesis-generating

Because this was a narrative review, no formal study-level risk-of-bias instrument was applied. **Table 2** was instead developed using a qualitative study-level appraisal. Design strength was determined from the study feature that most increased the reliability or clinical interpretability of the evidence, including randomization, prospective follow-up, sample size, use of an appropriate comparison group, direct physiological measurement, or clinically relevant outcomes. The main limitation was defined as the feature most likely to restrict causal inference, precision, or generalizability, including observational or cross-sectional design, small sample size, short follow-up, absence of an untreated comparator, residual confounding, or restriction to a specific population. The interpretation was formulated by considering the design strength and main limitation together and by limiting the conclusion to the level of inference supported by the study design. Accordingly, observational studies were used to support associations rather than causality, while randomized or longitudinal studies were interpreted in relation to treatment effects or temporal relationships when supported by their design. These principles were applied consistently across the studies summarized in **Table 2**, and no numerical score or formal ranking was assigned.

Throughout the manuscript, evidence was differentiated according to its source and the level of inference supported by the study design. Human clinical and physiological evidence was distinguished from observational associations, animal studies, cellular studies, and proposed mechanisms. Observational findings were described as associations unless the study design supported a stronger inference. Animal and cellular findings were used primarily to support biological plausibility, while mechanisms without direct human validation were described as proposed or hypothesis-generating.

## 3 Hypothyroidism and metabolic syndrome: definitions and epidemiological background

Hypothyroidism can be classified according to the level of thyroid dysfunction. Overt primary hypothyroidism is characterized by elevated TSH together with reduced free thyroxine (FT4), whereas subclinical hypothyroidism is characterized by elevated TSH with FT4 remaining within the reference range (10). Primary hypothyroidism accounts for most cases and is commonly associated with Hashimoto thyroiditis, thyroidectomy, radioiodine therapy, or medication exposure (19). Central hypothyroidism results from insufficient hypothalamic or pituitary stimulation of the thyroid gland. It is characterized by reduced FT4 accompanied by low or inappropriately normal TSH (20). TSH alone does not reliably identify central hypothyroidism, and FT4 should therefore be interpreted together with the clinical context when this condition is suspected.

Metabolic syndrome is defined by the clustering of five principal cardiometabolic abnormalities. These include elevated waist circumference, elevated triglycerides, reduced HDL-C, elevated blood pressure, and elevated fasting glucose (21, 22). Under the 2005 IDF definition, central obesity is required and must be accompanied by any two of the remaining four components (23, 24). NCEP ATP III defines metabolic syndrome when any three of the five components are present, without requiring central obesity (25). The 2009 harmonized definition also permits diagnosis when any three components are present and applies population-specific waist thresholds (26, 27). Relevant treatment for lipid abnormalities, elevated blood pressure, or elevated glucose may satisfy the corresponding component. PCOS is associated with insulin resistance but is not a diagnostic component of metabolic syndrome.

Recent evidence further supports an epidemiological association between metabolic syndrome and subclinical hypothyroidism. A 2024 meta-analysis found that metabolic syndrome was associated with higher odds of subclinical hypothyroidism, with an OR of 2.56 and a 95% CI of 1.44-4.55 (28). The individual components of metabolic syndrome were not consistently associated with subclinical hypothyroidism. The prevalence and clinical meaning of coexisting thyroid and metabolic abnormalities may also vary by age, sex, thyroid autoimmunity, and population context (29–32). Postmenopausal women represent one clinically relevant group because thyroid abnormalities frequently coexist with metabolic and cardiovascular risk factors (33).

## 4 The link between hypothyroidism and metabolic syndrome

### 4.1 The association between hypothyroidism and obesity

Hypothyroidism and obesity are clinically associated, although the relationship differs according to thyroid status (34, 35). Overt hypothyroidism can be accompanied by modest weight gain, but part of this change reflects sodium and water retention rather than increased adipose tissue. In a one-year controlled follow-up study, 12 patients with newly diagnosed hypothyroidism experienced a reduction in mean body weight from 83.7 to 79.4 kg after levothyroxine (LT4) treatment, while fat mass remained essentially unchanged (14). Weight reduction after correction of overt hypothyroidism may therefore reflect loss of excess body water to a considerable extent. Weight-related responses after LT4 are heterogeneous, and changes in appetite or food intake do not uniformly translate into fat loss (36).

Thyroid hormone deficiency can influence energy balance through lower resting energy expenditure and reduced thermogenesis (37, 38). Thyroid hormones participate in brown adipose tissue activity and regulate UCP1 expression, while reduced thyroid hormone action can decrease thermogenic capacity (39–42). These mechanisms provide a biological basis for altered energy expenditure in overt hypothyroidism, although their contribution to obesity varies among patients. The principal pathways are summarized in **Figure 1**.

**Figure 1:**
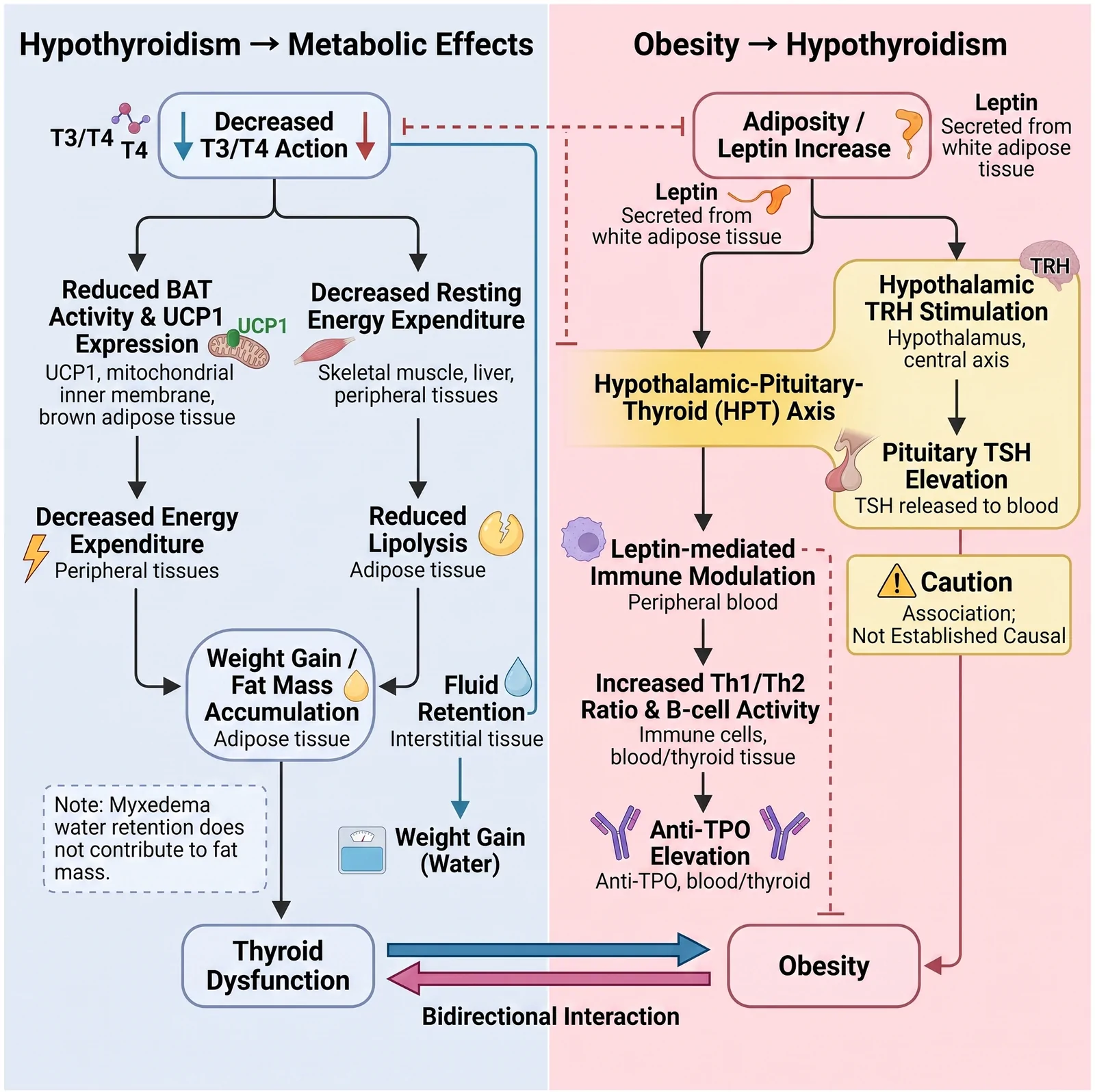
Mechanisms linking hypothyroidism and obesity. The figure is organized into two principal domains. The left column illustrates the established effects of thyroid hormone deficiency on energy balance. Overt hypothyroidism reduces resting energy expenditure and brown adipose tissue activity via decreased UCP1 expression, contributing to reduced thermogenesis and weight gain. A distinction is made between weight gain resulting from fluid retention (sodium and water accumulation) and that arising from adipose tissue accumulation, as these respond differently to levothyroxine treatment. The right column depicts the less certain reverse pathways through which obesity may influence thyroid function. Adiposity-associated increases in leptin can stimulate hypothalamic TRH and subsequent pituitary TSH secretion, leading to mild hyperthyrotropinemia. In parallel, obesity-related leptin changes are associated with thyroid autoimmunity markers (e.g., anti-TPO) through immune modulation, although this association does not establish causality. The central bidirectional arrow highlights the clinical overlap between the two conditions. Evidence level is indicated visually throughout the figure. Solid arrows denote pathways supported by human clinical or physiological evidence. Dashed arrows denote pathways supported primarily by animal or cellular studies. Observational relationships are labeled as associations rather than presented as established causal pathways. Proposed mechanisms without direct human validation are explicitly labeled as hypothesis-generating. Green arrows denote stimulation or activation, while red T-bars denote inhibition. BAT, brown adipose tissue; FT4, free thyroxine; T3, triiodothyronine; TRH, thyrotropin-releasing hormone; TSH, thyroid-stimulating hormone; UCP1, uncoupling protein 1; anti-TPO, anti-thyroid peroxidase antibodies.

The relationship is less straightforward in subclinical hypothyroidism. A meta-analysis of 22 observational studies found that obesity was associated with hypothyroidism overall, with an RR of 1.86 and a 95% CI of 1.63-2.11. The association was stronger for overt hypothyroidism, with an RR of 3.21 and a 95% CI of 2.12-4.86. For subclinical hypothyroidism, the corresponding RR was 1.70 with a 95% CI of 1.42-2.03 (35, 43, 44). These estimates indicate different strengths of association according to thyroid status and should be interpreted within the observational design of the included studies.

Obesity is frequently accompanied by modest differences in circulating TSH even when FT4 remains within the reference range (45, 46). In a cross-sectional analysis of 11,224 euthyroid adults, higher TSH within the reference range was associated with greater adiposity (47). Similar mild TSH elevations have been reported in individuals with morbid obesity (48). Leptin-mediated hypothalamic regulation may contribute to these changes by influencing TRH and TSH secretion (49–52). In this setting, modest hyperthyrotropinemia may reflect a potentially reversible alteration in hypothalamic-pituitary-thyroid-axis regulation rather than a defined thyroid hormone-resistance syndrome (53). Thyroid function should be interpreted together with FT4 and evidence of thyroid autoimmunity when mild TSH elevation is identified in patients with obesity.

Obesity is also associated with thyroid autoimmunity. A recent clinical study found higher serum leptin and TSH levels in individuals with obesity, while serum leptin was positively correlated with anti-TPO levels (43, 44, 54, 55). This association provides clinical support for a relationship between obesity-related leptin changes and thyroid autoimmunity but does not establish that obesity directly causes autoimmune thyroid disease.

Overall, current evidence supports an association between obesity and thyroid dysfunction but does not indicate a uniform causal relationship. Overt hypothyroidism can contribute to changes in body weight and energy expenditure, whereas the metabolic significance of mild TSH elevation is less certain when thyroid hormone levels remain normal. Weight loss may be accompanied by changes in TSH, but this observation is better interpreted as a dynamic relationship between adiposity and thyroid-axis regulation rather than proof of a single causal direction. Representative clinical evidence related to obesity and thyroid dysfunction is summarized in **Table 1**.

### 4.2 The association between hypothyroidism and dyslipidemia

Dyslipidemia is a common metabolic manifestation of hypothyroidism. Overt hypothyroidism is most consistently associated with increased total cholesterol and LDL-C, while triglycerides may also increase (56–59). The lipid phenotype of subclinical hypothyroidism is less uniform. In a study using repeated thyroid testing and strict diagnostic criteria, no independent association between persistent subclinical hypothyroidism and total cholesterol, LDL-C, or HDL-C was confirmed after adjustment for relevant confounding factors (60). Thyroid status should therefore be considered when the strength of this association is interpreted.

The mechanistic basis is best established for impaired cholesterol and lipoprotein metabolism. Reduced thyroid hormone action decreases hepatic LDLR-mediated clearance of circulating LDL-C (57, 61). Cholesterol elimination may also decline because thyroid hormone participates in the regulation of bile acid synthesis (62). Elevated TSH may contribute independently to hepatic lipid metabolism through effects on HMGCR and PCSK9, while altered lipoprotein lipase activity may promote triglyceride-rich lipoprotein accumulation (63, 64). Thyroid hormone also regulates hepatic fatty-acid handling and oxidative pathways (65–67). The principal pathways are summarized in **Figure 2**.

**Figure 2:**
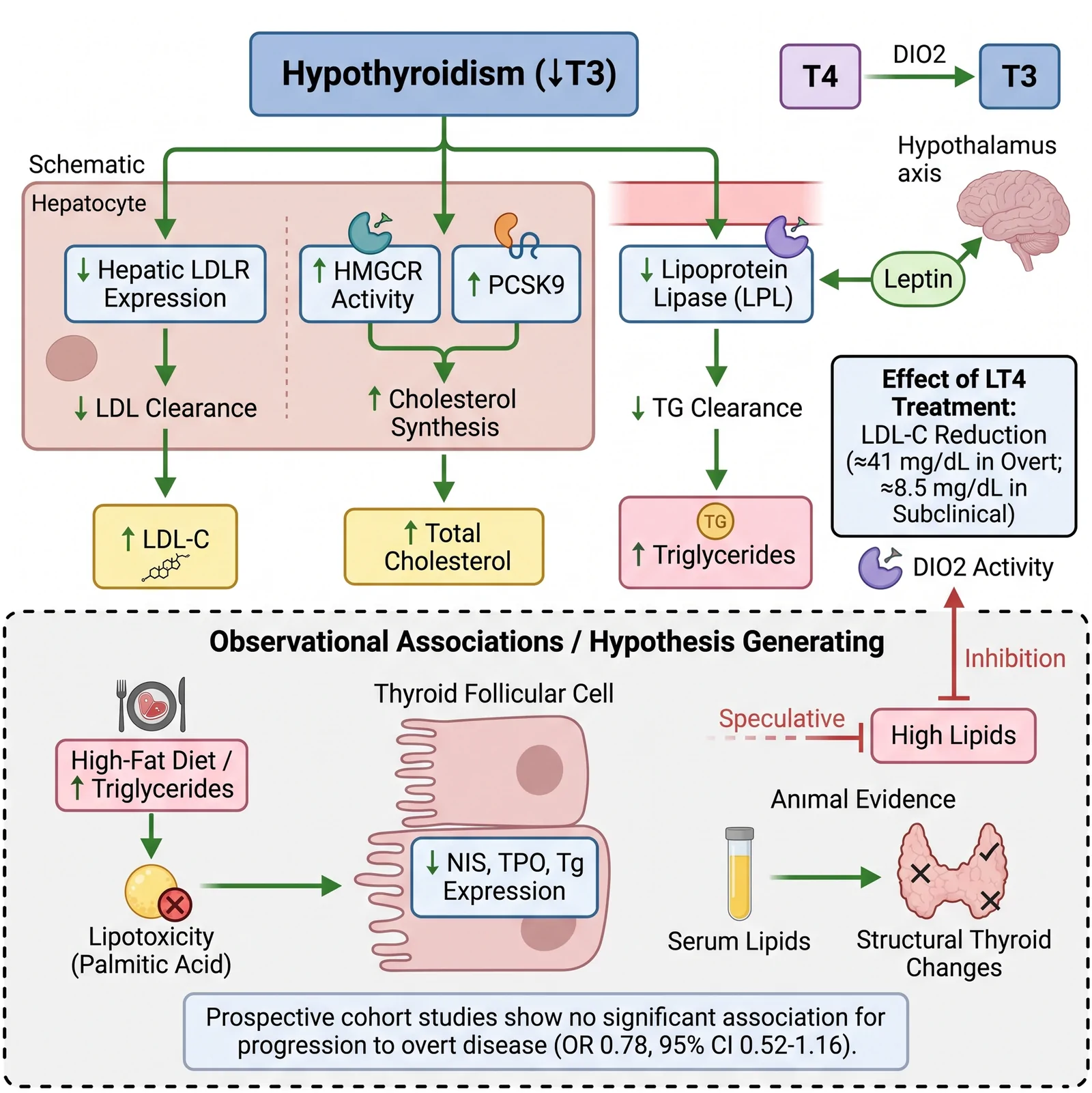
Mechanisms linking hypothyroidism and dyslipidemia. The figure is structured to reflect the relative strength of evidence for each direction. The upper portion summarizes the well-established effects of hypothyroidism on lipid metabolism. Reduced thyroid hormone action decreases hepatic LDL receptor-mediated clearance of LDL-C and increases cholesterol synthesis through upregulation of HMGCR and PCSK9. Impaired lipoprotein lipase activity further reduces triglyceride clearance. The quantitative impact of levothyroxine treatment on LDL-C reduction is shown for both overt and subclinical hypothyroidism. The lower portion presents the emerging and less certain hypothesis that lipid abnormalities may influence thyroid function. Observational associations between hypertriglyceridemia and subclinical hypothyroidism are shown together with experimental evidence from animal models of high-fat feeding. Cellular evidence involving palmitic acid-mediated downregulation of thyroglobulin, sodium iodide symporter, and thyroperoxidase is included as a potential lipotoxicity mechanism. In the T4-to-T3 conversion pathway, DIO2 is explicitly shown, and the speculative inhibition of DIO2 by lipid excess is indicated with a dashed red T-bar. Evidence level is indicated visually throughout the figure. Solid arrows denote pathways supported by human clinical or physiological evidence. Dashed arrows denote pathways supported primarily by animal or cellular studies. Observational relationships are labeled as associations rather than presented as established causal pathways. Proposed mechanisms without direct human validation are explicitly labeled as hypothesis-generating. A boxed annotation highlights that prospective cohort data have not confirmed a significant association between baseline metabolic syndrome and incident overt hypothyroidism. DIO2, type 2 iodothyronine deiodinase; HMGCR, 3-hydroxy-3-methylglutaryl-CoA reductase; LDL, low-density lipoprotein; LDL-C, low-density lipoprotein cholesterol; LDLR, LDL receptor; LPL, lipoprotein lipase; LT4, levothyroxine; NIS, sodium iodide symporter; PCSK9, proprotein convertase subtilisin/kexin type 9; TG, triglycerides; Tg, thyroglobulin; TPO, thyroid peroxidase.

Treatment findings provide additional support for the thyroid-to-lipid direction. A systematic review and guideline synthesis found that LT4 treatment in overt hypothyroidism reduced LDL-C by approximately 41.11 mg/dL, with a 95% CI of 35.69-46.53 mg/dL. The lipid response in subclinical hypothyroidism was smaller, and pooled randomized evidence showed an LDL-C reduction of approximately 8.51 mg/dL, with a 95% CI of 3.48-13.92 mg/dL (68–71). Correction of thyroid hormone deficiency can therefore improve the lipid profile, although persistent dyslipidemia should be assessed as a separate cardiovascular risk factor after euthyroidism is restored (72, 73).

Evidence that dyslipidemia may influence thyroid function remains substantially weaker than evidence for the effect of hypothyroidism on lipid metabolism. In a population-based case-control study, hypertriglyceridemia was associated with subclinical hypothyroidism in men with an OR of 1.325 and a 95% CI of 1.002-1.753. A similar association was observed in women with an OR of 1.397 and a 95% CI of 1.217-1.604 (18, 74). These findings identify an epidemiological association but do not establish the direction of causality.

Experimental studies provide additional biological plausibility. Rats exposed to a prolonged high-fat lard diet developed higher triglyceride concentrations in serum and thyroid tissue together with lower FT4 and higher TSH. Structural changes were also observed in the thyroid gland (74–76). High-fat feeding produces several metabolic alterations simultaneously, and these findings do not identify a single circulating lipid as the direct cause of thyroid dysfunction.

Cellular evidence suggests that specific fatty acids may affect thyroid hormone synthesis. In human primary thyrocytes, palmitic acid exposure reduced the expression of thyroglobulin, sodium iodide symporter, and thyroperoxidase. Iodide uptake and thyroperoxidase activity were also reduced (77). These findings support lipotoxicity as a potential mechanism linking lipid excess with altered thyroid function, but cellular responses cannot be directly extrapolated to clinical hypothyroidism or Hashimoto thyroiditis.

Taken together, current evidence supports a possible lipid-to-thyroid interaction but remains insufficient to establish dyslipidemia as a direct cause of hypothyroidism. A 2024 systematic review of prospective studies did not find a significant association between baseline metabolic syndrome and subsequent overt hypothyroidism, with an OR of 0.78 and a 95% CI of 0.52-1.16 (78). The reverse pathway should therefore be regarded as an emerging hypothesis, while longitudinal human studies are needed to determine whether particular lipid abnormalities independently influence thyroid function. The principal clinical evidence informing the thyroid-lipid relationship and its interpretive limitations are summarized in **Tables 1**, **2**.

### 4.3 The association between hypothyroidism and insulin resistance

Hypothyroidism is associated with alterations in insulin action and glucose metabolism, although the magnitude of these changes varies according to thyroid status and the metabolic characteristics of the study population (79–82). Peripheral glucose utilization is one of the better supported components. In a clinical study comparing overt hypothyroidism, subclinical hypothyroidism, and euthyroid controls, both hypothyroid groups showed higher HOMA-IR and lower Matsuda index values. Insulin-stimulated GLUT4 translocation and glucose transport were also reduced (12). The PI3K-AKT pathway participates in both insulin and thyroid hormone signaling and represents an important shared node in metabolic regulation (83–85). Alterations in these overlapping pathways may contribute to impaired peripheral glucose handling when thyroid dysfunction and insulin resistance coexist. The principal pathways are summarized in **Figure 3**.

**Figure 3:**
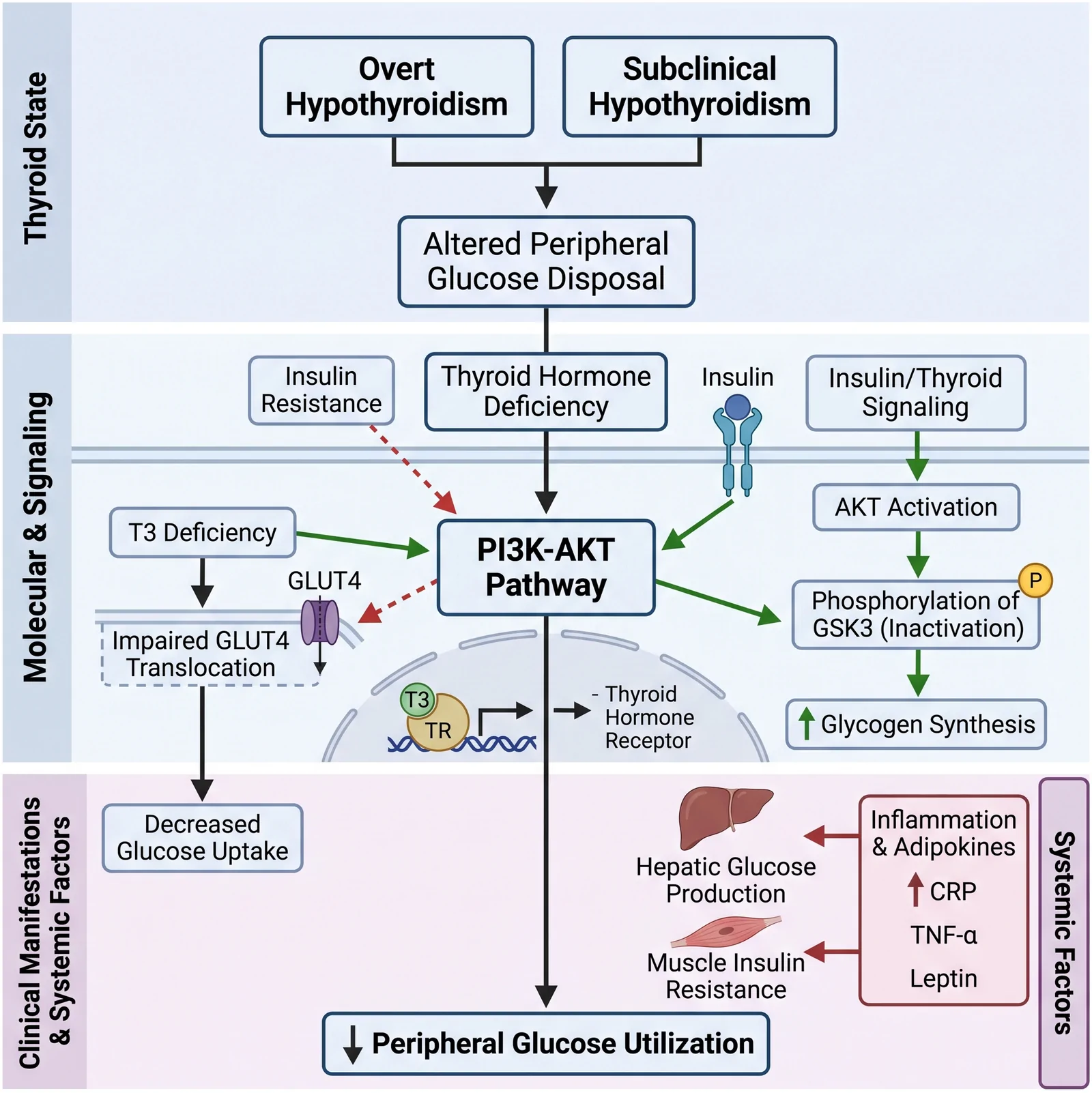
Mechanisms linking hypothyroidism and insulin resistance. The figure is organized as a hierarchical pathway from thyroid dysfunction (top) to molecular signaling nodes (middle) and downstream metabolic consequences (bottom). Overt and subclinical hypothyroidism are distinguished where evidence differs. The PI3K-AKT pathway is shown as a shared signaling node in both thyroid hormone and insulin action, rather than as a unidirectional causal chain from insulin resistance to impaired T3 signaling. The corrected GSK3 pathway is depicted as follows: AKT-mediated phosphorylation inactivates GSK3, which promotes glycogen synthesis (green arrow), correcting the previous inversion of this signaling logic. Reduced T3 action also impairs GLUT4 translocation, decreasing peripheral glucose uptake. Systemic factors are presented in a separate column, including inflammation-related markers (CRP, corrected from GRP), adipokines, and hepatic glucose output. Evidence level is indicated visually throughout the figure. Solid arrows denote pathways supported by human clinical or physiological evidence. Dashed arrows denote pathways supported primarily by animal or cellular studies. Observational relationships are labeled as associations rather than presented as established causal pathways. Proposed mechanisms without direct human validation are explicitly labeled as hypothesis-generating. The dotted separator distinguishes clinical syndromes from molecular signaling events and does not indicate evidence level. All abbreviations are expanded in the legend. The figure does not depict a self-sustaining “vicious cycle,” reflecting the current evidence base in which reverse effects of insulin resistance on thyroid function remain uncertain and hypothesis-generating. AKT, serine/threonine kinase; CRP, C-reactive protein; GLUT4, glucose transporter type 4; GSK3, glycogen synthase kinase 3; HOMA-IR, homeostatic model assessment of insulin resistance; IL-6, interleukin-6; PI3K, phosphoinositide 3-kinase; QUICKI, quantitative insulin sensitivity check index; T3, triiodothyronine; TNF-α, tumor necrosis factor-alpha; TR, thyroid hormone receptor.

Hepatic glucose metabolism is also influenced by thyroid status, although its specific contribution to insulin resistance in hypothyroidism is less clearly defined in humans. Thyroid hormones participate in the regulation of glucose turnover, while changes in thyroid status can modify the metabolic response to insulin (80, 84). In a glucose-clamp study of patients with hypothyroidism, thyroid hormone treatment increased basal glucose disposal and improved glucose disposal in relation to circulating insulin levels (86). These findings indicate altered glucose handling in hypothyroidism, while a uniform increase in hepatic glucose production cannot be assumed from the available clinical evidence. Higher circulating insulin concentrations may reflect compensatory secretion together with changes in insulin action, but fasting insulin alone cannot distinguish altered secretion from altered clearance. Cellular studies also indicate that thyroid status can influence proinsulin gene expression, although the clinical significance remains uncertain (87).

Low-grade inflammation and adipokine alterations may provide additional links between hypothyroidism and impaired insulin action. Patients with subclinical hypothyroidism have been reported to exhibit higher hs-CRP and fasting insulin concentrations, although HOMA-IR was not significantly different from euthyroid controls in the same study (88, 89). Changes in adipokines have also been described in thyroid dysfunction (90–93). These findings suggest that inflammatory and adipose-tissue signals may modify insulin sensitivity, but their contribution is influenced by adiposity and the underlying thyroid state. Inflammatory stress within autoimmune thyroid disease may also interact with insulin signaling (94). Ectopic lipid deposition in liver and muscle may further impair insulin action, particularly when hypothyroidism coexists with fatty liver disease (95–97).

Clinical studies support an association between hypothyroidism and insulin resistance, but the findings are heterogeneous. Population-based studies have identified relationships between thyroid function and insulin sensitivity (81, 98–100). Associations have also been reported when metabolic syndrome components or the triglyceride-glucose index are used to characterize metabolic risk (30–32, 79). More recent evidence indicates that the association may depend on baseline glucose metabolism. In a study including normoglycemic adults and patients with newly diagnosed type 2 diabetes, subclinical hypothyroidism was associated with higher insulin resistance in the normoglycemic population, while a comparable difference was not observed in the group with type 2 diabetes (16). The metabolic context should therefore be considered when the relationship between thyroid dysfunction and insulin resistance is interpreted.

The effect of LT4 replacement on insulin sensitivity is also inconsistent across clinical studies. A glucose-clamp study reported improved insulin-related glucose disposal after thyroid hormone replacement (86). Small studies in subclinical hypothyroidism have also reported improvements in selected insulin-sensitivity measures (101, 102). In contrast, a more recent study of untreated primary hypothyroidism found lower fasting insulin after three months of LT4 treatment but no significant improvement in HOMA-IR or QUICKI (17). Restoration of euthyroidism may improve selected metabolic parameters without consistently normalizing insulin resistance. Persistent abnormalities in glucose metabolism should be assessed independently after thyroid function has been corrected.

Evidence for a reverse effect of insulin resistance on thyroid dysfunction remains more limited. Metformin treatment has been associated with lower TSH in some patients with treated hypothyroidism and type 2 diabetes (103, 104), whereas a randomized trial in subclinical hypothyroidism did not demonstrate a significant TSH reduction (15). Human studies have also reported altered adipocyte DIO2 expression in obesity without a corresponding association with systemic insulin sensitivity (105, 106). Current findings support interaction between metabolic and thyroid regulation, while the clinical contribution of insulin resistance to the development or progression of hypothyroidism remains uncertain. Insulin resistance should be treated according to metabolic indications, while hypothyroidism should be managed according to thyroid-specific criteria. Representative clinical studies examining thyroid function, insulin resistance, and related treatment effects are summarized in **Table 1**.

## 5 Treatment and clinical management strategies

Clinical management should first distinguish thyroid hormone replacement from treatment of accompanying metabolic abnormalities. Overt primary hypothyroidism should be treated with LT4 as standard replacement therapy. The starting dose should be individualized according to age, body size, cardiovascular status, and the severity of thyroid dysfunction. For most nonpregnant adults, treatment should aim to maintain TSH within an appropriate laboratory- and age-specific reference range rather than below a fixed threshold of 2.5-3.0 mIU/L. A TSH reference range of approximately 0.4-4.0 mIU/L is generally used for replacement therapy (69, 73, 107).

Different treatment targets apply in selected clinical settings. During pregnancy and preconception, LT4 treatment should follow pregnancy-specific recommendations and corresponding TSH targets (108). In central hypothyroidism, TSH is not a reliable marker for dose adjustment, and treatment should instead be guided primarily by FT4 (20). Patients with differentiated thyroid cancer may require disease-specific TSH management according to oncological status and response to treatment (109).

Levothyroxine remains the standard thyroid hormone replacement therapy for primary hypothyroidism. Liothyronine is not routinely recommended because its shorter pharmacokinetic profile can produce substantial fluctuations in serum T3 after administration and may increase symptoms or adverse effects associated with thyroid hormone excess in susceptible patients (107). Current clinical trials have not consistently demonstrated superior outcomes with LT4 and LT3 combination therapy compared with LT4 monotherapy (110). Alternative thyroid hormone preparations should therefore not be used routinely in place of LT4.

Management of subclinical hypothyroidism requires a more selective approach. A TSH level at or above 10 mIU/L provides a stronger basis for considering LT4 treatment, whereas treatment below this threshold should remain individualized according to symptoms, thyroid autoimmunity, age, cardiovascular risk, and the broader clinical context (10, 111, 112). TSH may contribute to risk stratification but should not be used as an isolated treatment target. In an individual-participant analysis of 55,287 adults, the hazard ratio for coronary heart disease events was 1.00 with a 95% CI of 0.86-1.18 at TSH 4.5-6.9 mIU/L and 1.17 with a 95% CI of 0.96-1.43 at 7.0-9.9 mIU/L. The corresponding hazard ratio increased to 1.89 with a 95% CI of 1.28-2.80 at TSH 10-19.9 mIU/L (11).

In subclinical hypothyroidism, LT4 should be prescribed according to thyroid-specific indications rather than with the expectation of broadly reversing metabolic abnormalities. A small clinical study reported improved insulin sensitivity after LT4 treatment (101, 102). However, a more recent study found lower fasting insulin after treatment but no significant improvement in HOMA-IR or QUICKI (17). Restoration of euthyroidism may therefore improve selected metabolic parameters without consistently normalizing insulin resistance. Persistent dyslipidemia or abnormal glucose metabolism should be assessed separately and treated according to the corresponding metabolic indications. Representative interventional evidence and its principal limitations are summarized in **Tables 1**, **2**.

Obesity should be managed as a concurrent metabolic condition rather than by increasing thyroid hormone replacement beyond the dose required to achieve euthyroidism. Weight management should be individualized according to obesity severity and associated complications. Structured lifestyle intervention remains fundamental, while pharmacological treatment or metabolic surgery may be considered when clinically indicated (113, 114). Thyroid hormone replacement should not be intensified solely for the purpose of weight reduction.

Dyslipidemia requires coordinated assessment of thyroid status and cardiovascular risk. In overt hypothyroidism, the lipid profile should be reassessed after euthyroidism has been restored because total cholesterol and LDL-C frequently improve following LT4 treatment (57, 68, 70, 71). Persistent dyslipidemia should then be managed according to established cardiovascular risk and lipid-lowering indications. In subclinical hypothyroidism with clinically relevant hyperlipidemia, thyroid hormone treatment may be considered in selected patients, although the expected lipid benefit is generally smaller (73).

Prediabetes and diabetes should be managed according to established metabolic indications. Structured lifestyle intervention remains central to delaying progression from prediabetes to diabetes, while metformin may be considered in patients with a higher risk of progression (114, 115). In type 2 diabetes, glucose-lowering therapy should be individualized according to glycemic goals, body weight, cardiovascular or renal conditions, and treatment burden (116). Metformin, GLP-1 receptor agonists, and SGLT2 inhibitors should be selected for their established metabolic indications rather than for presumed thyroid-specific effects. Metformin may also be considered for metabolic outcomes in adults with PCOS according to PCOS-specific recommendations (117). Potential thyroid effects of GLP-1 receptor agonists remain under study and should not determine routine use (118). The coexistence of hypothyroidism does not itself provide an additional indication for these medications.

Potential drug interactions should be considered during long-term LT4 treatment. Calcium and iron preparations can reduce LT4 absorption and should be separated from LT4 administration. Other medications or gastrointestinal factors that impair absorption may also require adjustment of administration timing. TSH should generally be reassessed approximately 4–6 weeks after LT4 initiation or a dose change, followed by periodic monitoring after a stable dose has been achieved (107). FT4 should also be assessed when TSH does not adequately reflect the clinical setting.

Monitoring should address thyroid and metabolic targets in parallel. Body weight, blood pressure, lipid profile, and glycemic status should be followed according to the corresponding metabolic condition and treatment strategy. Patients with several concurrent abnormalities may benefit from coordinated care involving thyroid management, nutritional support, weight management, and cardiometabolic risk assessment (3). This approach allows each condition to be treated according to its established clinical indications while maintaining consistent therapeutic goals.

## 6 Future development and research directions

Future observational research should prioritize longitudinal cohorts with repeated assessment of thyroid and metabolic status. Overt primary hypothyroidism and subclinical hypothyroidism should be analyzed separately because their metabolic associations and treatment implications are not equivalent. Treated and untreated participants should also be distinguished to avoid attributing therapy-related changes to the natural course of thyroid dysfunction. Baseline thyroid autoimmunity and iodine status should be recorded to permit evaluation of their contribution to subsequent thyroid changes. Prespecified analyses should further examine variation by age, sex, ethnicity, and population setting. The limitations summarized in **Table 2** further support the need for longitudinal cohorts and adequately powered subgroup-specific trials.

Repeated thyroid measurements are particularly important when body weight changes substantially. Measurements obtained before and after weight loss or weight gain may help determine whether obesity-associated changes in TSH persist as adiposity changes (45, 48, 53). Longitudinal datasets should also be used to examine temporal relationships among thyroid status and metabolic abnormalities. Causal-mediation approaches may help determine whether changes in adiposity, inflammation, or insulin resistance contribute to the observed associations while reducing reliance on cross-sectional correlations.

Future studies should determine whether intervention strategies require further stratification in populations with distinct clinical risks. Older adults may require more cautious LT4 initiation and dose adjustment because excessive replacement can increase cardiovascular and skeletal risks (107). This issue is particularly relevant to postmenopausal women, in whom thyroid abnormalities often coexist with metabolic and cardiovascular risk factors (33). Randomized evidence has not demonstrated a clear cardiovascular benefit of LT4 in older adults with subclinical hypothyroidism (13). Future trials should therefore identify patients who are most likely to benefit while minimizing the risk of overtreatment.

Family history represents a different consideration because first-degree relatives of patients with Hashimoto thyroiditis have a substantially increased risk of developing the disease (119). Prospective studies should determine whether targeted thyroid function testing and autoimmune assessment improve early identification in this population. Monitoring intervals should be adjusted according to subsequent changes in thyroid function rather than using family history alone as an indication for treatment.

Future LT4 trials should focus on clinically defined subgroups rather than treating subclinical hypothyroidism as a uniform condition. TSH range, age, thyroid autoimmunity, iodine status, treatment history, and the underlying cardiometabolic phenotype should be prespecified. Outcomes should extend beyond normalization of thyroid laboratory values. Patient-reported symptoms, quality of life, metabolic control, treatment-related adverse effects, progression to overt hypothyroidism, and cardiovascular events should be evaluated (13, 72, 73, 120). Longer follow-up is needed to determine whether treatment produces patient-centered benefit in subgroups that are not adequately represented in current trials.

The effects of metabolic medications on thyroid function should be evaluated separately from their established metabolic benefits. Future studies should define the clinical indication, dose, duration, and treatment exposure before thyroid-related outcomes are assessed. Drug-specific analyses are preferable to a generalized combined-treatment model because different agents may affect thyroid measurements through distinct pathways (104, 118). This issue is particularly relevant to metformin, for which randomized studies have not demonstrated a consistent effect on TSH (15). Prospective trials should distinguish changes in thyroid laboratory measurements from clinically meaningful changes in thyroid disease and should evaluate adverse effects together with potential benefits.

## 7 Conclusion

Overall, hypothyroidism and metabolic syndrome are clinically associated through overlapping abnormalities in body weight, lipid metabolism, glucose regulation, and inflammatory signaling. Current evidence is more consistent for the effects of hypothyroidism on metabolic phenotypes than for a reverse effect of metabolic syndrome on thyroid function. Metabolic abnormalities may influence thyroid-axis regulation or peripheral thyroid hormone metabolism, but the clinical magnitude and causal relevance of these effects remain uncertain.

Clinical management should address thyroid dysfunction and metabolic abnormalities according to their respective indications. LT4 remains the standard replacement treatment when hypothyroidism requires therapy (10). Persistent obesity, dyslipidemia, or abnormal glucose metabolism should be evaluated independently after thyroid status has been addressed and should receive appropriate metabolic management (68). In subclinical hypothyroidism, the degree of TSH elevation can contribute to risk assessment, but treatment decisions should not rely on TSH alone.

Further longitudinal and interventional studies are needed to clarify the direction of these associations and to identify patients who may benefit from more individualized management. Until such evidence is available, the relationship between hypothyroidism and metabolic syndrome is best interpreted as a clinically relevant association with several shared and potentially interacting pathways rather than as an established self-sustaining causal cycle.
